# Numerical Analysis on Fatigue Crack Growth at Negative and Positive Stress Ratios

**DOI:** 10.3390/ma16103669

**Published:** 2023-05-11

**Authors:** Abdulnaser M. Alshoaibi, Yahya Ali Fageehi

**Affiliations:** Mechanical Engineering Department, Jazan University, Jazan 45142, Saudi Arabia; yfageehi@jazanu.edu.sa

**Keywords:** fatigue crack propagation, linear elastic fracture mechanics, negative stress ratios, equivalent stress intensity factor, von Mises stress, fatigue life cycles

## Abstract

The finite element method was used to investigate the effect of the stress ratio on fatigue crack propagation behavior within the framework of the linear elastic fracture mechanics theory. The numerical analysis was carried out using ANSYS Mechanical R19.2 with the unstructured mesh method-based separating, morphing, and adaptive remeshing technologies (SMART). Mixed mode fatigue simulations were performed on a modified four-point bending specimen with a non-central hole. A diverse set of stress ratios (*R* = 0.1, 0.2, 0.3, 0.4, 0.5, −0.1, −0.2, −0.3, −0.4, −0.5), including positive and negative values, is employed to examine the influence of the load ratio on the behavior of the fatigue crack propagation, with particular emphasis on negative *R* loadings that involve compressive excursions. A consistent decrease in the value of the equivalent stress intensity factor ($\Delta K_{eq}$) is observed as the stress ratio increases. The observation was made that the stress ratio significantly affects both the fatigue life and the distribution of von Mises stress. The results demonstrated a significant correlation between von Mises stress, $\Delta K_{eq}$, and fatigue life cycles. With an increase in the stress ratio, there was a significant decrease in the von Mises stress, accompanied by a rapid increase in the number of fatigue life cycles. The results obtained in this study have been validated by previously published literature on crack growth experiments and numerical simulations.

## 1. Introduction

Most metallic materials experience significant amounts of stable crack growth under cyclic loading preceding catastrophic failure. In some circumstances, fatigue loads may become essential and even cause catastrophic failures, which might result in significant financial losses or even fatalities. Thus, in order to prevent such substantial damage, it is essential to compute the 3D fatigue crack propagation behavior and the allowable life span of cracked components. The stress ratio (*R*) is calculated as the ratio of the minimum stress to the maximum stress in each loading cycle. According to experimental data, fatigue crack growth (FCG) depends on both the stress ratio, $R=\sigma_{\min}/\sigma_{\max}$, and the range of stress intensity factors, Δ*K* [1,2,3]. Experimental evidence shows that under cyclic loading with a constant stress ratio, an increase in the applied stress intensity factor range results in a higher rate of crack propagation (da/dN), and plastic strain accumulation takes place at the crack front [4,5]. The accumulation of plastic strain and the dissipation of plastic energy at the crack tip both increase when the load ratio decreases [6]. A common crack propagation model is the Paris–Erdogan law. The problem of fatigue crack propagation was addressed through the theory of linear elastic fracture mechanics (LEFM) [7,8,9,10]. To appropriately assess fatigue crack growth, numerous factors should be considered, such as the level of stress, frequency of the load, load ratio, and type of material. It is generally known that the load ratio has an effect on the growth of fatigue cracks and threshold behavior. In fatigue testing, a negative stress ratio indicates that the specimen experiences more compressive stress cycles than tensile stress cycles during each loading cycle. In other words, the minimum stress in the loading cycle is a compressive stress, and the maximum stress is a tensile stress. Studies incorporating negative stress ratios have received little attention despite the importance of FCG research. Furthermore, inconsistent results have been observed. The potential effect of the negative component of cyclic stress on crack growth is a major issue [6,11,12,13]. Most often, there is no differentiation between negative and positive stress ratios in the literature [14]. Numerous investigations have shown that material’s fatigue failure is significantly influenced by the level of the applied stress [15,16,17,18]. In accordance with LEFM, the level of stresses caused by a remote load near the crack front are evaluated using the stress intensity factors (SIFs). The finite element method (FEM) is the preferred computational approach for modeling damage and failure under fatigue loading due to its ability to provide solutions for stress intensity factors, displacement, stress, and strain for various types of problems. From a numerical modeling perspective, the fatigue crack propagation is now addressed by a variety of software, including FRANC3D [19] ABAQUS [20], ANSYS [21,22,23,24,25,26], ZENCRACK [27], COMSOL [28], BEASY [29], and NASTRAN [30].

Numerous experimental models have been reported for crack growth analysis [31,32,33], but they are both time-consuming and costly. As a result, in order to save laboratory time, costs, and effort, a precise numerical method is essential for a fatigue crack growth investigation. Using ANSYS Mechanical R19.2 software, this investigation computed the equivalent stress intensity factors and corresponding fatigue life of the modified four-point bend specimen with a single non-central hole under different stress ratios. Despite its importance, the analysis of fatigue crack growth data under negative stress ratios is usually carried out in a way that is similar to that under positive stress ratios, with insufficient attention devoted to this particular issue [34]. The objective of this study is to enhance comprehension of this concern and raise awareness among readers about the significant uncertainties in evaluating fatigue life at negative stress ratios while also illustrating the correlation between stress ratios and stress intensity factor, fatigue life, and von Mises stress. The ANSYS software was used to investigate these effects using a modified four-point bending specimen.

## 2. Computational Analysis Using ANSYS

This study utilized ANSYS Mechanical R19.2, a robust and powerful finite element analysis software tool, to calculate mixed-mode stress intensity factors (SIFs), crack growth path, stress distribution, and fatigue analysis for the modified four-point bending specimen. By using the unstructured mesh strategy, which reduces long preprocessing periods and allows for decreased meshing time, SMART meshes the finite element model with a higher-order SOLID187 tetrahedral element. In order to incorporate modifications in the crack front caused by crack propagation, the meshes were updated automatically at each step of the solution. As the crack propagates, it applies a localized remesh algorithm rather than the enrichment method. During the solution part of the analysis, the SIFs were determined using interaction integral evaluation. In the field of fracture mechanics, the fatigue crack propagation angle is a crucial parameter that can significantly affect the durability and safety of engineering structures. The maximum tangential stress criterion, which is a widely used approach, is employed to determine the fatigue crack propagation angle [35,36,37,38]. The direction formula of crack propagation in ANSYS are as follows [21,39]:

(1) $$\theta =\cos^{-1}\;\left(\frac{3K_{II}^{2}+K_{I}\sqrt{K_{I}^{2}+8K_{II}^{2}}}{K_{I}^{2}+9K_{II}^{2}}\right)$$

where *K_I_* and *K_II_* denotes the opening mode and in-plane shear mode of SIF, respectively.

The equivalent stress intensity factor range ($\Delta K_{eq}$) was employed for fatigue life prediction in ANSYS Mechanical’s SMART crack growth tool. In order to ensure the safe and reliable operation of engineering structures, it is essential to assess their damage tolerance, particularly in cases where they are subjected to cyclic loading. Therefore, accurate assessment of the fatigue life is necessary for designing and maintaining structures to meet their expected service life and safety requirements. Using a modified Paris law formula, Tanaka [40] proposed a power law that correlates fatigue crack propagation with an equivalent stress intensity factor. The formula is expressed as follows:

(2) $$\frac{da}{dN}=C{\left(\Delta K_{eq}\right)}^{m}$$

where *a* represents the crack length, *N* is the fatigue life cycles, and *C* and *m* are the Paris constant and Paris exponent, respectively. The formula for $\Delta K_{eq}$ is the following [39,41]:

(3) $$\Delta K_{eq}=\frac{1}{2}\cos\left(\frac{\theta }{2}\right)\left[\Delta K_{I}(1+\cos\theta )-3\Delta K_{II}\sin\theta \right]$$

where:

(4) $$\begin{array}{l} \Delta K_{I}=K_{I}^{\max}-K_{I}^{\min}=(1-R)K_{I}^{\max}\\ \Delta K_{II}=K_{II}^{\max}-K_{II}^{\min}=(1-R)K_{II}^{\max} \end{array}$$

and *R* is the stress ratio.

Equation (2) can be solved to calculate the total number of cycles for a given crack growth increment (∆*a*), which can be expressed as follows:

(5) $$\int_{0}^{\Delta a}\frac{da}{C{\left(\Delta K_{eq}\right)}^{m}}=\int_{0}^{\Delta N}dN=\Delta N$$

## 3. Numerical Implementation

### Modified Four-Point Bending Specimen with One Hole

**Figure 1:**
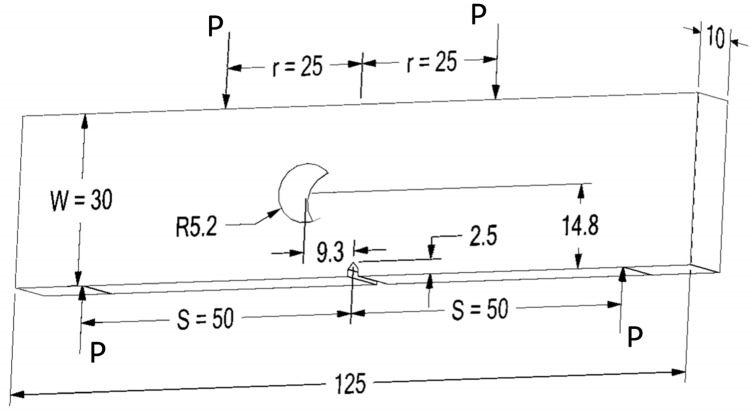
Modified four-point bending specimen.

**Figure 2:**
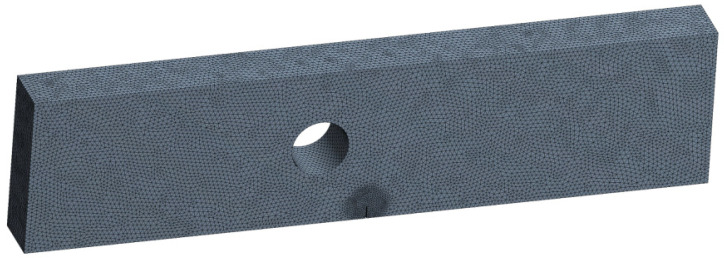
First mesh generated by Ansys.

A four-point bending load is applied to a single edge notched bend specimen with a 5.2 mm diameter hole radius and an eccentricity of 9.3 from the notch line, as illustrated in Figure 1. The simulations were performed using the same specimen size, crack size, and loading conditions as in the experiment reported in [42]. A constant amplitude fatigue loading with a load ratio of *R* = 0.1 was applied to this geometry, and an applied load of *P* = 10 kN. Figure 2 displays the initial mesh for this geometry, which had 259,254 nodes and 176,151 elements with a 1 mm element size. SAE 1020 carbon steel was utilized in the study, and its mechanical properties are presented in Table 1.

**Figure 3:**
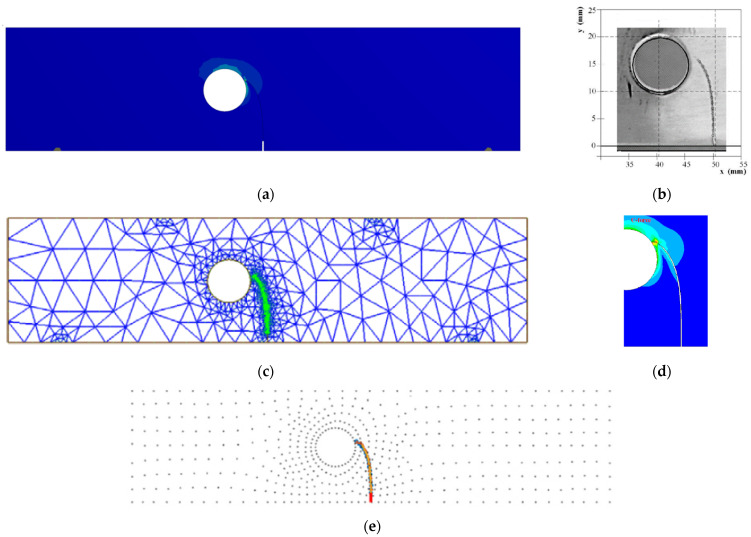
Crack propagation path (**a**) Ansys, (**b**) experimental trajectory [42], (**c**) numerical [43], (**d**) numerical [44], (**e**) numerical [45].

The crack propagation path calculated by the ANSYS software agrees very well with the experimental trajectory observed by [42], and it can be matched to estimated paths achieved by further simulation approaches, such as the FEM based on local Lepp–Delaunay obtained by [43], the FEM with configurationally forces obtained by [44], and the extended meshfree-smoothed technique obtained by [45], as displayed in Figure 3a–e, respectively. In the beginning, the crack tip is quite far from the hole so that the crack grows in a straight line. The presence of a hole cause the crack trajectory to deviate from its initial path and change direction towards the hole. The presence of a hole in a material can significantly affect the behaviour of a propagating crack. Due to this influence, the crack trajectory may deviate from its initial path and change direction towards the hole. This phenomenon is a well-known effect in fracture mechanics, and it is often referred to as crack deflection. The change in the direction of the crack propagation can have significant implications for the structural integrity of engineering components, and it is crucial to consider this effect when designing and analyzing structures that contain holes or defects.

The SIF is a crucial parameter for evaluating fatigue life. The analytical solution for the opening mode of SIFs in a standard four-point bending geometry without a hole is expressed as [46]:

(6) $$K_{I}=\bar{\Delta K}\frac{6P(s-r)\sqrt{\pi a}}{W^{2}t}$$

where $\bar{\Delta K}$ is the normalized stress intensity factor, *W* denotes the width, *t* denotes the thickness, *P* denotes the load, *s* and *r* are the distances between the upper and lower supports identified in Figure 1, and *a* denotes the crack length. The normalized SIF for the standard specimen without a hole was expressed as [46]:

(7) $$\bar{\Delta K}=\frac{1.1215}{{\left(1-\frac{a}{W}\right)}^{\left(3/2\right)}}\left[\frac{5}{8}-\frac{5}{12}(a/W)+\frac{1}{8}{\left(a/W\right)}^{2}+5{\left(a/W\right)}^{2}{\left(1-\frac{a}{W}\right)}^{6}+\frac{3}{8}\exp(-6.1342(a/W)/(1-\frac{a}{W})\right]$$

Equation (7) is no longer valid for computing the normalized SIF in the holed specimen as a result of the geometry modification. The calculated SIF for a modified four-point bending specimen using ANSYS may be fitted using a sixth degree polynomial to obtain the formula given:

(8) $$\bar{\Delta K}=\;\mathrm{1819.8}{\left(a/W\right)}^{6}\;-\;\mathrm{3079.1}{\left(a/W\right)}^{5}\;+\;\mathrm{2085.7}{\left(a/W\right)}^{4}\;-\;\mathrm{708.13}{\left(a/W\right)}^{3}\;+\;\mathrm{127.85}{\left(a/W\right)}^{2}\;-\;\mathrm{10.452}(a/W)\;+\;\mathrm{1.3715}$$

**Figure 4:**
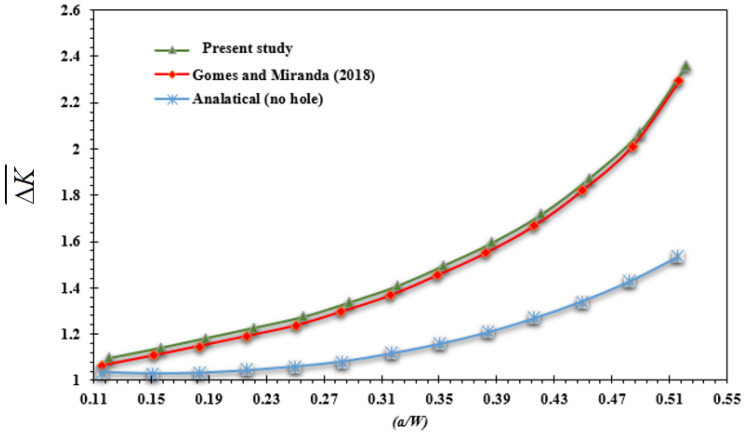
Normalized SIF for the modified and standard specimen [42].

Figure 4 compares the results for the normalized stress intensity factor computed by ANSYS for a crack length of 15.5 mm to results from the numerical investigation obtained by Gomes and Miranda [42] by the boundary element method for the modified geometry with a hole, as well as the reference solution provided in Equation (2) for the standard specimen without a hole. This demonstrates how normalized SIF values are significantly affected by the hole’s position.

The estimated values of the opening mode of SIFs match very well with the numerical data reported by Gomes and Miranda [42] using ABAQUS and BemCracker2D, respectively, as displayed in Table 2.

**Figure 5:**
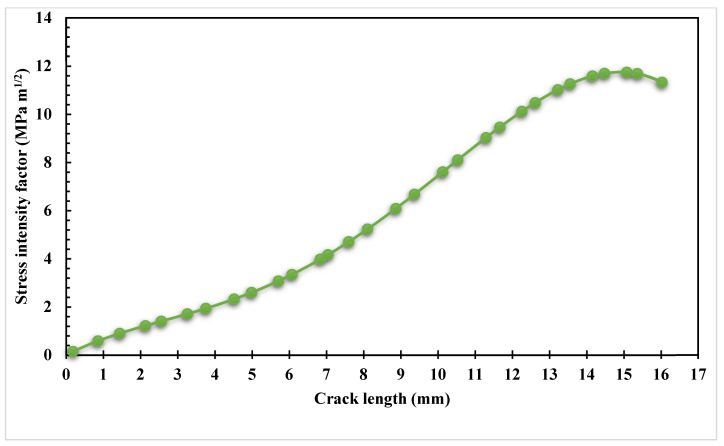
The first mode of stress intensity factor, *K_I_*.

**Figure 6:**
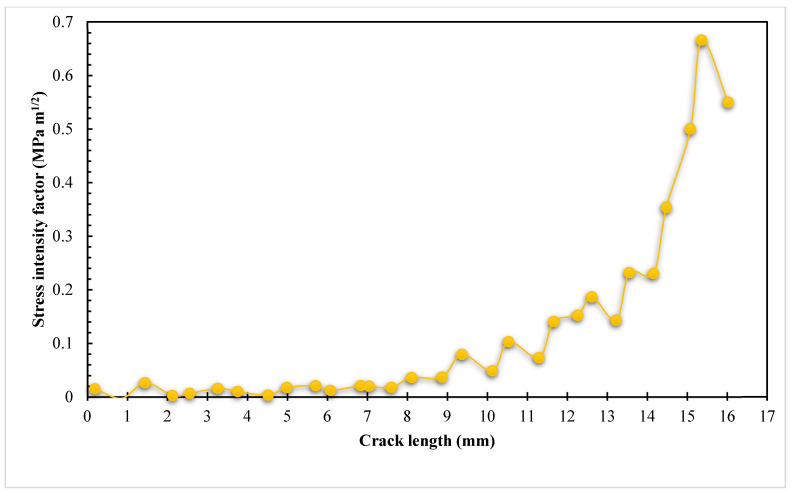
The second mode of stress intensity factor, *K_II_*.

The estimated values of the *K_I_* and *K_II_* modes of SIFs are shown in Figure 5 and Figure 6 below. These figures show that when *K_I_* dominates the crack propagation trajectory, the crack begins to grow on a straight path. When a hole influences a crack’s direction, the crack grows in the direction of the hole and modifies its path as the value of *K_II_* rises beyond the crack length of 6 mm to a maximum value of 0.66 MPa m^0.5^.

**Figure 7:**
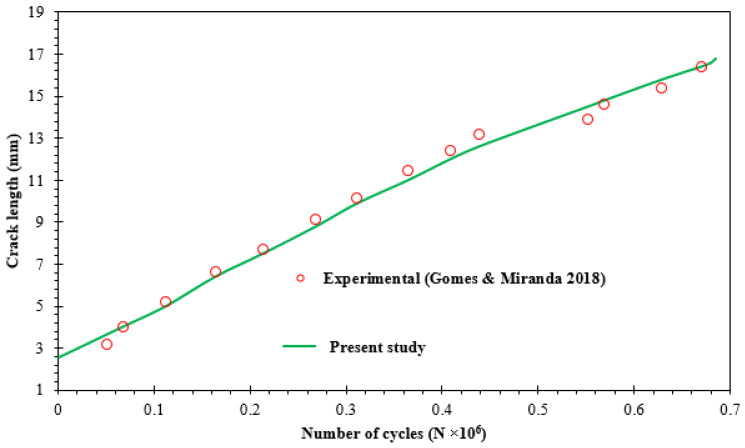
Fatigue life compared to experimental data [42].

Figure 7 compares the Gomes and Miranda’s experimental data [42] with estimated fatigue life cycles of the present analysis. The present results and the experimental data showed a strong correlation for the fatigue life cycles.

**Figure 8:**
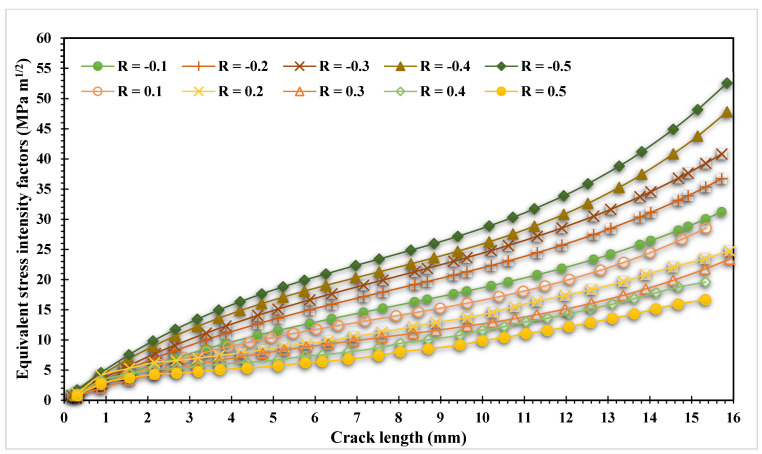
Equivalent range of SIF for positive and negative stress ratio.

The constant amplitude equivalent stress intensity factor range at various stress ratios, both positive and negative, determined numerically using ANSYS for the studied geometry are shown in Figure 8 for the ten simulations. The data presented in this figure provides important insights into the relationship between the stress ratio and equivalent stress intensity factor in fatigue crack propagation. Specifically, the figure shows that as stress ratio increases, there is a corresponding decrease in the equivalent stress intensity factor, indicating that higher stress ratios require less energy to facilitate crack propagation than lower stress ratios. This information is critical in predicting the fatigue life of engineering components subjected to cyclic loading and can help guide decisions related to their design, maintenance, and safety.

**Figure 9:**
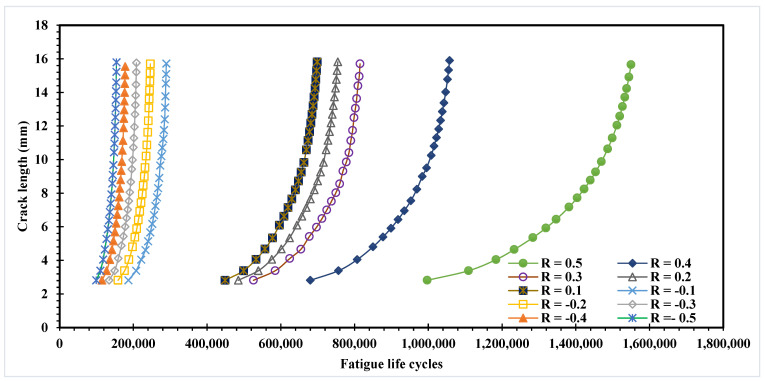
Fatigue life cycles under various stress ratios.

**Figure 10:**
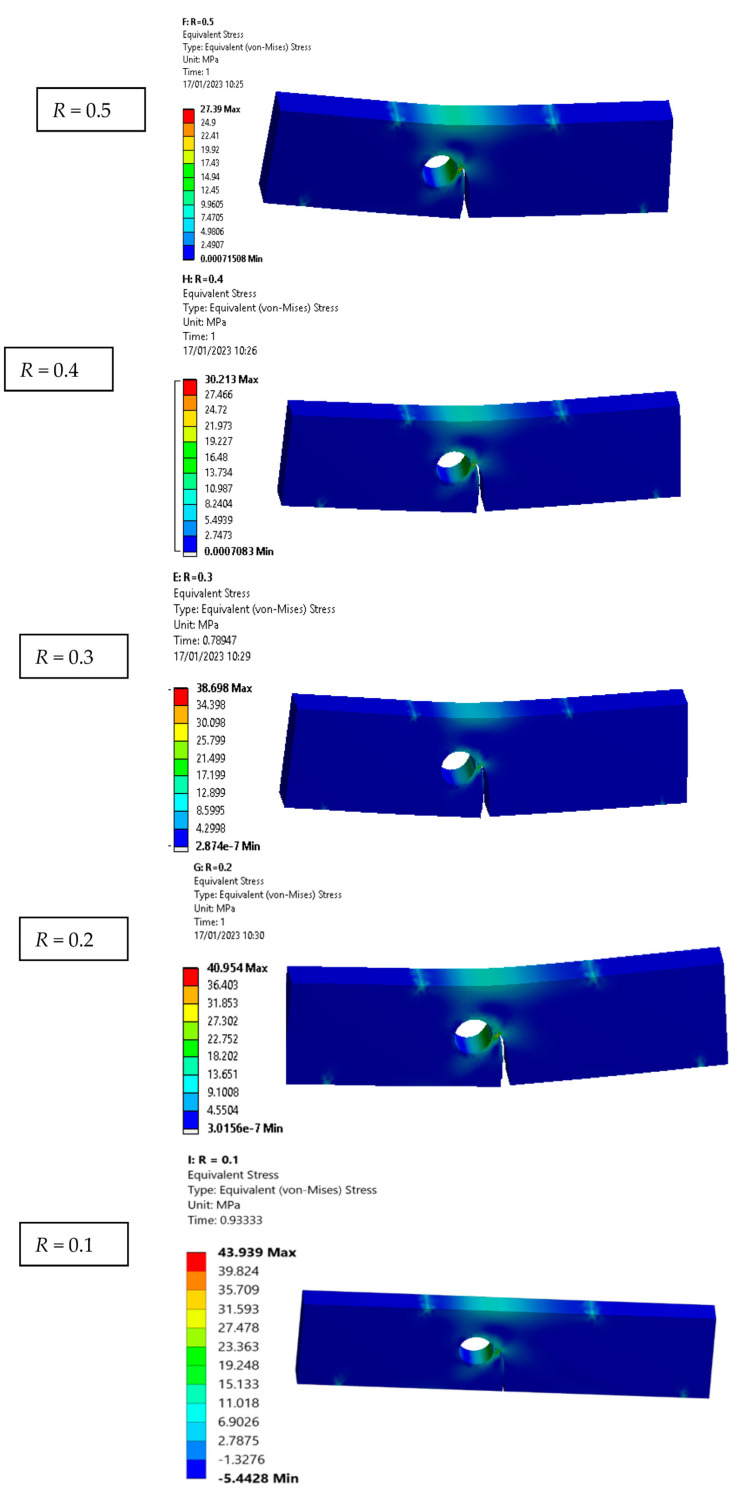

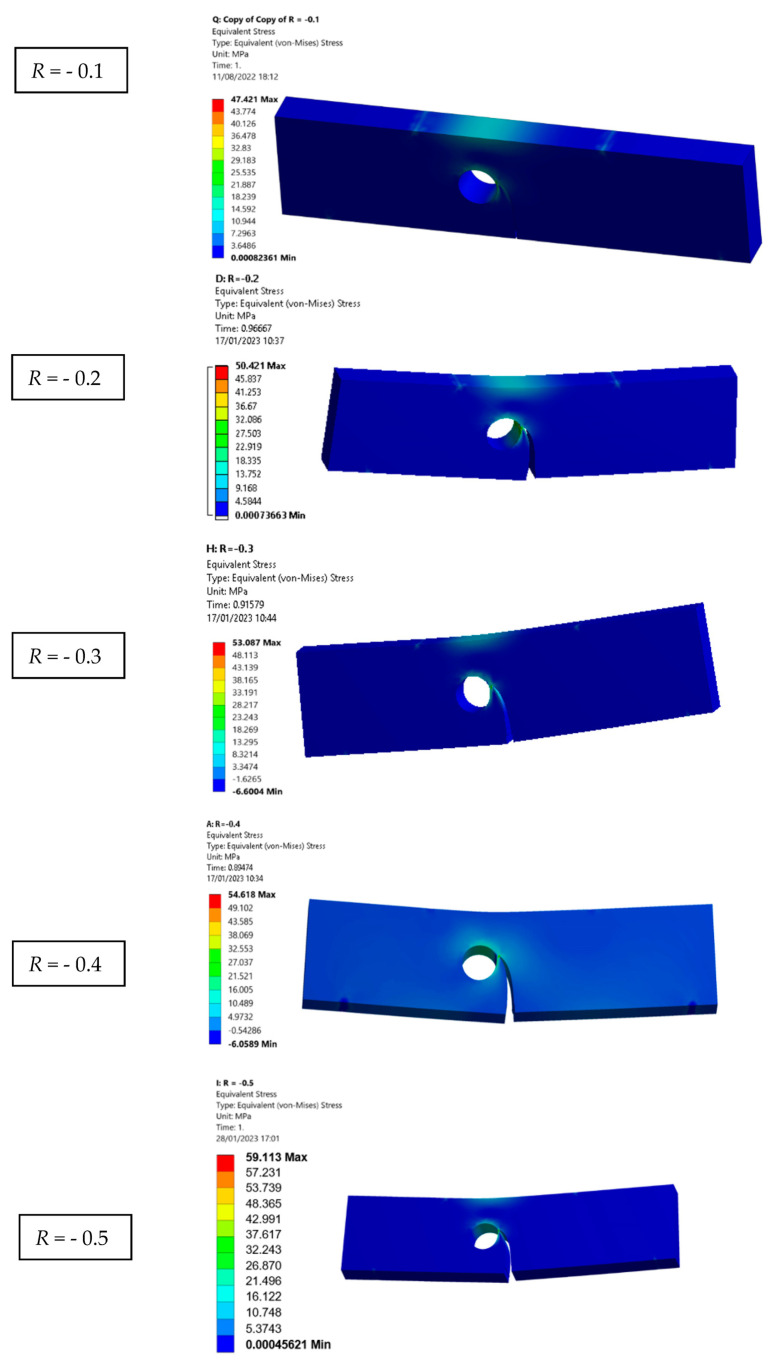
Distribution of Von Mises stress for various stress ratios.

The relationship between fatigue life cycles and stress ratios is an important consideration in the design and analysis of mechanical components that experience cyclic loading. The stress ratio affects the fatigue life of a component because it influences the magnitude and direction of the cyclic loading that the component experiences. When the stress ratio is positive, the minimum stress value during each cycle is greater than zero, meaning that the material experiences a net tensile load during each cycle. Conversely, when the stress ratio is negative, the minimum stress value during each cycle is less than zero, meaning that the material experiences a net compressive load during each cycle. Table 3 displays the fatigue life cycles: *N_f_* for the modified four-point bending specimen under various stress ratios. Figure 9 depicts the fatigue life curves with respect to the crack length for different stress ratios. It is observable that when the stress ratio decreases, the number of cycles decreases. Table 3 and Figure 9 indicate that a component exposed to a positive stress ratio will experience a shorter fatigue life compared to a component exposed to a negative stress ratio. This is due to the fact that a positive stress ratio results in a net tensile load during each cycle, which leads to a faster propagation of fatigue cracks. On the other hand, a negative stress ratio results in a net compressive load during each cycle, causing less damage to the material and resulting in a longer fatigue life. To estimate the distribution of stress and the location of stress concentration, the von Mises stress is considered. Contour plots of von Mises stresses are very useful to give a sense of the stress distribution. Due to a reduction in von Mises stresses, which were evidently depicted in Figure 10, the components’ life increases as the plastic zone’s size enlarges. The data presented in this figure reveal an interesting relationship between the von Mises stress and stress ratio in fatigue crack propagation. Specifically, the figure shows that as stress ratio decreases from 0.5 to −0.5, there is an increase in von Mises stress. Interestingly, these results align with earlier studies that have reported a lack of a significant influence of the stress ratio on crack propagation trajectory under constant amplitude loading [12,47].

## 4. Conclusions

In this study, ANSYS Mechanical R19.2 was utilized to perform finite element analyses and investigate the effect of stress ratios on fatigue crack propagation. Specifically, the study focused on examining the fatigue life cycles and equivalent stress intensity factor of a modified four-point bending specimen under varied stress ratios. Specifically, the study focused on assessing the equivalent stress intensity factor, fatigue life cycles, and von Mises stress under stress ratios ranging from R = 0.1 to −0.5. By examining these variables across a range of stress ratios, the study aimed to provide a comprehensive understanding of the effects of the stress ratio on fatigue crack propagation and contribute to the development of more durable and reliable engineering structures subjected to cyclic loading.

The investigation presented in this study led to several significant conclusions. First, it was observed that an increase in the negative stress ratio led to an increase in the equivalent stress intensity factor. This finding emphasizes the importance of considering the stress ratio when designing components for cyclic loading, as a more negative stress ratio can lead to more severe damage and, ultimately, failure.

Second, reducing the stress ratio was found to lead to a decrease in fatigue life, indicating a shorter lifespan for the material under cyclic loading. This emphasizes the importance of selecting an appropriate stress ratio to ensure the durability of components and structures.

Third, the study found that reducing the magnitude of von Mises stresses can lead to an increase in fatigue life cycles. This finding has significant implications for engineers and designers, as it suggests that by reducing the magnitude of von Mises stresses, the durability and lifespan of engineering structures can be enhanced, potentially improving safety and reliability in various industries.

Finally, the study found that the stress ratio did not have a significant effect on the trajectory of the crack propagation. This observation suggests that factors such as geometry and material properties may have a more significant influence on the behavior of fatigue crack growth.

## Figures and Tables

**Table 1:** Mechanical properties of the simulated specimen.

Property	Metric Unit Value
Modulus of elasticity, *E*	205 GPa
Poisson’s ratio, υ	0.29
Yield strength, *σ_y_*	285 MPa
Ultimate strength, *σ_u_*	491 MPa
Threshold stress intensity factor, *K*_th_	$11.6\;\mathrm{MPa}\sqrt{m}$
Paris’ law coefficient, *C*	8.59 × 10^−14^ mm/(cycle MPa mm^0.5^)
Paris law exponent, *m*	4.26

**Table 2:** Results of *K_I_* using ANSYS, ABAQUS [42], and BemCracker2D [42].

	*K_I_* (MPa m^0.5^)		
Crack Tip Distance (mm)	Present Study	ABAQUS [42]	BemCracker2D [42]
2.5	1.475	1.47	1.46
6.4	2.625	2.62	2.63
12.2	9.632	9.628	9.77

**Table 3:** Fatigue life at positive and negative stress ratios.

Stress Ratio (*R*)	Fatigue Crack Growth Life, *N_f_* (Cycles)
0.1	6.97 × 10^5^
0.2	7.52 × 10^5^
0.3	8.15 × 10^5^
0.4	1.05 × 10^6^
0.5	1.55 × 10^6^
−0.1	2.89 × 10^5^
−0.2	2.46 × 10^5^
−0.3	2.08 × 10^5^
−0.4	1.78 × 10^5^
−0.5	1.45 × 10^5^

## Data Availability

All relevant data are contained in the present manuscript.
